# Retraction: Increased Age-Adjusted Cancer Mortality After the Third mRNA-Lipid Nanoparticle Vaccine Dose During the COVID-19 Pandemic in Japan

**DOI:** 10.7759/cureus.r143

**Published:** 2024-06-26

**Authors:** Miki Gibo, Seiji Kojima, Akinori Fujisawa, Takayuki Kikuchi, Masanori Fukushima

**Affiliations:** 1 Primary Health Care, Matsubara Clinic, Kochi, JPN; 2 Pediatrics, Nagoya Pediatric Cancer Fund, Nagoya, JPN; 3 Cardiovascular Medicine, Honbetsu Cardiovascular Medicine Clinic, Honbetsu, JPN; 4 Translational Research & Health Data Science, Learning Health Society Institute, Nagoya, JPN

The Editors-in-Chief have retracted this article. Upon post-publication review, it has been determined that the correlation between mortality rates and vaccination status cannot be proven with the data presented in this article. As this invalidates the conclusions of the article, the decision has been made to retract.

The authors disagree with this retraction.

